# Supplementary material for: MetaRibo-Seq measures translation in microbiomes
Source: Nat Commun. 2020 Jun 29;11:3268. doi: 10.1038/s41467-020-17081-z (PMC7324362; doi:10.1038/s41467-020-17081-z)
Supplement: Supplementary file 10 — Supplementary Data 7 [file 41467_2020_17081_MOESM10_ESM.zip › File2/Confidence_High_Taxonomy/137132_out.krona.html]

Javascript must be enabled to view this page.

members
magnitude
magnitudeUnassigned
count
unassigned
taxon
rank

137132\_out

2

2
2
superkingdom

1239
2
phylum

2
186801
class

order
186802
2

species
1

SRS063190\_contig\_number\_contig-100\_2957.96718
1897043

family
541000
1

genus
1
216851

species
1

SRS016335\_contig\_number\_43711
853
